# Compensatory hemodynamic changes in response to central hypovolemia in humans: lower body negative pressure: updates and perspectives

**DOI:** 10.1007/s10974-022-09635-z

**Published:** 2022-11-15

**Authors:** Nandu Goswami

**Affiliations:** 1 Division of Physiology, Gravitational Physiology and Medicine Research Unit, Otto Löwi Research Center of Vascular Biology, Inflammation, and Immunity, Medical University of Graz, Neue Stiftingtalstrasse 6, D-5, 8036 Graz, Austria; 2 College of Medicine, Mohammed Bin Rashid University of Medicine and Health Sciences, Dubai, United Arab Emirates

**Keywords:** Hemodynamics, Sex, Hormones, Blood pressure, Heart rate, Tolerance

## Abstract

Central hypovolemia is accompanied by hemodynamic compensatory responses. Understanding the complex systemic compensatory responses to altered hemodynamic patterns during conditions of central hypovolemia—as induced by standing up and/or lower body negative pressure (LBNP)—in humans are important. LBNP has been widely used to understand the integrated physiological responses, which occur during sit to stand tests (orthostasis), different levels of hemorrhages (different levels of LBNP simulate different amount of blood loss) as well as a countermeasure against the cephalad fluid shifts which are seen during spaceflight. Additionally, LBNP application (used singly or together with head up tilt, HUT) is useful in understanding the physiology of orthostatic intolerance. The role seasonal variations in hormonal, autonomic and circulatory state play in LBNP-induced hemodynamic responses and LBNP tolerance as well as sex-based differences during central hypovolemia and the adaptations to exercise training have been investigated using LBNP. The data generated from LBNP studies have been useful in developing better models for prediction of orthostatic tolerance and/or for developing countermeasures. This review examines how LBNP application influences coagulatory parameters and outlines the effects of temperature changes on LBNP responses. Finally, the review outlines how LBNP can be used as innovative teaching tool and for developing research capacities and interests of medical students and students from other disciplines such as mathematics and computational biology.

## Introduction

We subject our body to orthostatic challenge every time we stand up (Blaber et al. 2011; Goswami et al. 2015). During active standing, however, the exact potential of the compensatory responses of cardiovascular system may not be revealed. During intensive stress situations (e.g. exercise, diving, disease states or microgravity), the cardiovascular system shows its full potential and reveals cardiovascular regulatory features of physiological systems that otherwise might not be observable. Under certain occasions, there could be a decrease in cerebral perfusion to critical levels thus leading to syncope (Gao et al. 2008; Goswami et al. 2012). Intensive central hypovolemia can be experimentally induced via passive head up Tilt (HUT) and/or lower body negative pressure (LBNP) or a combination of both HUT + LBNP (Arbeille and Herault 1998; Cvirn et al. 2012). Negative suction pressure application on the lower body via a seal at anterior superior iliac spine causes caudal shifts in the blood and leads to a decreased venous return, and consequently, central hypovolemia (Baisch 1993; Balldin et al. 1996; Goswami et al. 2009a, b, c). During central hypovolemia, inhibitory effects of arterial and cardiopulmonary receptors on the central sympathetic system are removed leading to increased sympathetic activity (Barcroft et al. 1944), which ensure adequate cerebral perfusion and maintenance of blood pressure.

Some of the uses of LBNP include draining blood away from diseased organs, as a cardiovascular deconditioning prevention countermeasure (Batzel et al. 2009), for the study of hemorrhage induced responses (Cvirn et al. 2019). It is used in orthopedic rehabilitation, as it creates footward loading (detailed in Goswami et al. 2008, 2019a, b). In spaceflight, LBNP has been used to counter the headward shift of body fluids and to load the musculoskeletal system in spaceflight as it occurs on Earth. LBNP has been used for evaluation of responses to different therapeutic agents as well as to varying perturbations (e.g. across seasons and/or varying skin temperatures) and to investigate how the LBNP-induced responses vary across the sexes. For detailed effects of the LBNP on cardiovascular reflexes, please see (Bevegard et al. 1977; Bennett 1987; Balldin et al. 1996; Beske et al. 2001; Goswami et al. 2008, 2019a, b).

## Systemic effects of LBNP

LBNP is usually, though not always, applied to persons in the supine position, without skeletal muscle activity to minimize motion artifacts (Bennett 1987; Baisch 1993; Balldin et al. 1996). It can also be applied in exercising subjects (Hargens et al. 1991). Moreover, as this procedure does not require body position changes, it isolates the reflexes associated with change in the posture from supine to upright standing and also removes movement artifacts from the hemodynamic recordings.

Understanding cardiovascular responses to orthostatic challenge is largely based on the measurement of cardiac output, heart rate and lower limb venous compliance responses. LBNP elicits reproducible reflexive hemodynamic responses which maintain adequate heart and cerebral perfusion (Blaber et al. 2013) during the caudal fluid shifts. In comparing LBNP with centrifuge exposure for selecting pilot candidates, it was reported that the latter was more stressful than LBNP and that LBNP could be used as a convenient screening test to assess tolerance to central hypovolemia (Blaber et al. 2013).

Additional effects of the LBNP on different systems include on cerebral, autonomic, coagulation, pulmonary systems and hormones (Bevegard et al. 1977; Bennett 1987; Bertinieri et al. 1988; Berdeaux et al. 1992; Baisch 1993; Balldin et al. 1996; Beske et al. 2001). For instance, forearm muscle sympathetic activity doubles at − 60 mmHg LBNP (Haditsch et al. 2015; Roessler et al. 2011; Verma et al. 2017; Patel et al. 2016). Specific neurohormonal responses during LBNP are as follows (Blomqvist and Stone 1983): Adrenomedullin, adrenocorticotropic hormone (ACTH), prolactin and multifunctional neuropeptides such as galanin and atrial natriuretic peptide (ANP) rise as the central hypovolemia increases. Hypothalamic-pituitary components, such as vasopressin, are subsequently elevated with some delay, followed by several fold increases at presyncope (Hinghofer-Szalkay et al. 2011).

### LBNP and its potential effects on coagulation

LBNP application leads to hemoconcentration and elevations in blood viscosity and plasma proteins. That is, LBNP shifts the blood towards a more prothrombotic state (Zaar et al. 2009, 2014; van Helmond et al. 2015). Applying − 30 mmHg LBNP to healthy participants for about 10 min was shown to increase thrombin anti-thrombin (TAT) complex levels to those that occur during deep venous thrombosis (Zaar et al. 2009). Another study compared the effects of − 45 mmHg LBNP with blood loss of up to 1000 ml and concluded that coagulation changes are similar in both cases (van Helmond et al. 2015). The authors speculated that perhaps it was the accompanying elevations in sympathetic output—seen both in LBNP and bleeding—which could increase the tendency towards clot formation (372). This is not surprising, since it is known that trauma/ rupture to a vessel is accompanied by an increase in thrombosis, which, consequently, reduces exsanguination risk (van Helmond et al. 2015).

Even an increase in coagulation has been seen in healthy participants as they develop presyncopal signs and symptoms during LBNP (Cvirn et al. 2012). Cvirn et al. reported that the LBNP induced changes in hemoconcentration, blood viscosity and the plasma volume losses, and the increase in vasopressin at presyncope, could potentially contribute towards increases in endothelial activation markers (tissue factor, TF, and tissue plasminogen activator, TPA) as well as in thrombin generation parameters (thrombin-antithrombin complexes and prothrombin fragments 1 and 2). As vasopressin levels have been shown to be associated with increases in platelet aggregation and agglutination (Zaar et al. 2014), it could contribute significantly towards the increased coagulation seen at presyncope (Filep and Rosenkranz 1987).

## Aspects to consider when using LBNP

In addition to the types of LBNP protocol used (e.g. step vs ramp protocol), following aspects must be considered when applying LBNP:

### Magnitude and duration of LBNP suction application

Depending on the magnitude of suction, LBNP induced vascular pooling in the legs leads to decreases in venous return, central venous pressure, and stroke volume (Baisch 1993; Balldin et al. 1996). The onset and the degree of hypotension during central hypovolemia induced by LBNP also varies between participants’ (Bennett 1987; Baisch 1993; Balldin et al. 1996). It has been reported that − 20 mmHg LBNP application for 5 min leads to 500–1000 ml fluid displacement towards the peripheral vascular compartments (Baisch 1993; Balldin et al. 1996).

LBNP also causes an increase in total peripheral resistance; this is dependent on the magnitude of LBNP applied. Low levels of LBNP cause increases in sympathetic tone leading to vasoconstriction in the forearm and in the splanchnic area (Blaber et al. 2013). High LBNP levels (e.g. − 60 mmHg) lead primarily to renal vasoconstriction, decreases in glomerular filtration rate, renal plasma flow and urine production (see Baisch 1993; Balldin et al. 1996).

### LBNP magnitude and its influence on regional blood flows

It has been reported that LBNP above − 20 mmHg leads to differential blood distribution across the body compartments (detailed in Baisch 1993; Balldin et al. 1996)). Additionally, changing the LBNP from − 20 to − 40 mmHg causes leg volume to more than double; this leg pooling occurs exclusively in the venous vasculature. It has also been reported that at − 50 mmHg LBNP, women show larger pelvic blood pooling as compared to men (Bronzwaer et al. 2017).

The splanchnic vascular bed, which constitutes a major blood reserve, provides majority of regional vascular conductance, which helps in blood pressure maintenance central hypovolemia (Blaber et al. 2013; Hinghofer-Szalkay et al. 2008). LBNP of − 50 mmHg leads to a up to 1/3rd reduction in blood flow in the splanchnic area. In addition, due to the accompanying increased sympathetic activity, splanchnic blood flow continues to reduce as suction pressure is elevated (Brown et al. 1966).

### LBNP sealing location

LBNP induced responses depend also the position of the seal application. The LBNP sealing on its own modulates distribution of blood volume and perfusion of the liver even before commencement of the suction (Goswami et al. 2009a, b, c). During application of LBNP, different effects are seen when the LBNP seal is applied at different locations. For instance, when LBNP is applied at the upper abdomen—which includes the splanchnic region in the suction area—in contrast to the default LBNP seal at Iliac crest, larger drops in central blood volume and splanchnic blood flow occur. Upper sealing position also leads to greater compensatory elevations in heart rate (Goswami et al. 2009a, b, c).

### Influence of confounding variables

When carrying out LBNP studies, aspects related to subject selection criteria (e.g. sex, height, age, fitness levels), environmental conditions (e.g. seasons, temperature of the skin, time of the day, fasting or non-fasting state), and in-laboratory conditions (room temperature, humidity, noise) should be considered.

### Influence of temperature changes on LBNP responses

As changes in environmental temperature lead to changes in the generalized systemic responses, it is not surprising that LBNP tolerance is influenced by changes in temperature. Just as greater incidence of collapse and syncope during the hot seasons is reported (Goswami et al. 2017), exposure to heat decreases the LBNP tolerance while cold stress has been reported to increase it (Crandall 2000). LBNP tolerance, however, varies considerably between persons exposed to heat (Lee et al. 2013). Several factors such as skin temperature and/or venous compliance changes could contribute towards differences in LBNP tolerance during different temperature changes. These are now discussed in detail:

*Skin temperature* could influence both reflex vasoactive state of the skin and the direct local control in the skin (Wolthuis et al. 1974). Local changes in temperature on the skin could also influence the tolerance times (Charkoudian et al. 2002). For instance, heat application activates the cutaneous vasodilator system in resting humans and reduces LBNP tolerance while skin cooling improves LBNP tolerance (Wolthuis et al. 1974; Shibasaki et al. 2006). Skin cooling has been reported to be associated with increased sympathetic activity (Durand et al. 2004) and central venous pressure (Cui et al. 2005) as well as peripheral vasoconstriction.

*Venous compliance* changes during alterations in temperature, and the potential reductions in cerebral perfusion, have been postulated as contributing factors to reduced LBNP tolerance (Tripathi et al. 1984; Tripathi and Nadel 1986; Wilson et al. 2006). A study assessed Frank-Starling relationship with temperature changes and it was observed that heating leads to a downward shift of the operating point to a steeper portion of a Frank-Starling curve while cooling was associated with an upward shift to a flatter portion of the curve (Crandall 2000; Wilson et al. 2009). The Frank-Starling relationship during LBNP could also be influenced by heart rate, venous return, peripheral resistance and compliance (Wilson et al. 2009). The effects of whole-body heating and cooling on cardiovascular responses are presented in greater details in the elegant review of Wilson and Crandall (2011).

## LBNP as a teaching tool

Not only is LBNP a useful tool for demonstrating hemodynamic responses to central hypovolemia to students (Goswami et al. 2011, 2013), the recorded responses can be used for teaching optimization and physiological control loops to students from other disciplines, such as mathematics (Batzel et al. 2012; Etter el al. 2011). However, a big limitation of this measurement technique is that a careful monitoring of the volunteers during LBNP suction application is required. Not only is there a wide variability in the tolerance to LBNP application (Goswami et al. 2009a, b, c) but developmental of presyncopal signs and symptoms—a termination criteria for the study—can occur anytime in any of the volunteers (Goswami et al. 2021).

We have, therefore, recently investigated an innovative teaching approach: the “dry lab” approach (Goswami et al. 2021). In laboratories where there is no LBNP box available or there is lack of medical staff to monitor the study participants, a “dry lab” activity can be employed. This activity involves providing students with a review paper on LBNP, with which students can understand the usage of LBNP, identify issues/ confounding variables when carrying out LBNP experiments, and also how to interpret data from LBNP studies. For the latter activity, students are presented with hemodynamic data that have been collected during LBNP application. For instance, during graded LBNP (Blaber et al. 2013), across the sexes (Cvirn et al. 2019), at presyncope (Grasser et al. 2009a, b), and with interventions (e.g. mental arithmetic: Goswami et al. 2013); heat application (Wilson and Crandall 2011), amongst others.

Overall, it appears that our “dry lab” activity using LBNP to teach physiology exposes students even early in their medical studies and across disciplines to the basics of systems physiology and introduces them to experimental research. For example, providing insight into research includes formulation of a research question, designing (including, consideration of confounding variables), conducting, implementing and interpreting experimental data.

## Conclusions and recommendations

LBNP induces responses (cardiovascular and hormonal), which vary widely across persons. When carrying out LBNP studies, confounding variables such as selection criteria, LBNP protocol to be used and the laboratory- and environmental—conditions should be carefully accounted for. The call out box summarizes the potential changes in coagulation that could occur during LBNP as well as how temperature changes during LBNP application influence the physiological responses.

**Table:** 

*LBNP induced effects on coagulation* Hemoconcentration and plasma volume losses Elevations in blood viscosity and plasma proteins which predispose towards a pro-coagulant state Increases in endothelial activation markers (tissue factor, TF, and tissue plasminogen activator, TPA) as well as in thrombin generation parameters (thrombin-antithrombin complexes and prothrombin fragments 1 and 2) Increases in platelet aggregation and agglutination Accompanying elevations in sympathetic output—seen both in LBNP and bleeding—which could increase the tendency towards clot formation in both LBNP and bleeding *Effects of temperature changes on LBNP induced responses* LBNP tolerance is influenced by changes in temperature Heat decreases the LBNP tolerance while cold stress increases it Local changes in temperature on the skin could also influence the tolerance times Heat application activates the cutaneous vasodilator system and reduces LBNP tolerance while skin cooling improves LBNP tolerance *Venous compliance* changes during alterations in temperature *Decreases in cerebral blood flow during increased temperature*	
